# Different methods for resolving overlapping UV spectra of combination medicinal dose forms of ciprofloxacin and metronidazole

**DOI:** 10.1186/s13065-023-01007-z

**Published:** 2023-10-10

**Authors:** Mahmoud M. Sebaiy, Sobhy M. El-Adl, Alaa Nafea, Samar O. Aljazzar, Eslam B. Elkaeed, Amr A. Mattar, Samar S. Elbaramawi

**Affiliations:** 1 Medicinal Chemistry Department, Faculty of Pharmacy, Zagazig University, Zagazig, 44519 Egypt; 2 Department of Chemistry, College of Science, Princess Nourah bint Abdulrahman University, P.O. Box 84428, Riyadh 11671, Saudi Arabia; 3 Department of Pharmaceutical Sciences, College of Pharmacy, AlMaarefa University, Riyadh 13713, Saudi Arabia; 4 Pharmaceutical Medicinal Chemistry Department, Faculty of Pharmacy, Egyptian Russian University, Badr City, Cairo, 11829 Egypt

**Keywords:** Ciprofloxacin, Metronidazole, Advanced absorbance subtraction (AAS), Bivariate, Ratio difference, Spectrum subtraction

## Abstract

Four simple, specific, easy, precise and accurate spectrophotometric methods were developed for the first time to examine ciprofloxacin and metronidazole in combination, without having been separated beforehand by the developed methods. Ciprofloxacin and metronidazole were determined by utilizing advanced absorbance subtraction (AAS), spectrum subtraction, bivariate and ratio difference methods. Precision, repeatability, robustness, and accuracy were all determined to be within acceptable levels after each of these procedures underwent validation in accordance with ICH recommendations. Each method’s benefits and drawbacks are illustrated, and the proposed and reported methodologies were statistically compared.

**Supplementary Information:**

The online version contains supplementary material available at 10.1186/s13065-023-01007-z.

## Introduction

Ciprofloxacin (CIP); 1-cyclopropyl-6-fluoro-4-oxo-7-piperazin-1-ylquinoline-3-carboxylic acid (Additional file 1: Fig. S1) is a widely used fluoroquinolone with a wide range of medicinal applications. Its widespread use is due to the presence of multi-resistant bacteria that are exclusively responsive to ciprofloxacin. The currently available clinical evidence points to this medication's potentially increased efficacy. In the management of several nosocomial and community-acquired illnesses, including urinary tract infections, respiratory tract, and skin [1]. Additionally, ciprofloxacin is utilized to treat anthrax, certain types of plague and sexually transmitted diseases [2].

Metronidazole (MET); 2-(2-methyl-5-nitroimidazol-1-yl)ethanol; hydrochloride. Additional file 1: Fig. S1 is an antibiotic that is employed in the treatment of infections of the vaginal tract, liver, stomach, skin, joints, heart, brain and spinal cord, lungs, and bloodstream caused by bacteria. Metronidazole is also used to treat trichomoniasis, a parasite-based sexually transmitted illness. Even if one sexual partner has no symptoms, it is typical to treat both at once [2].

Ciprofloxacin which has a lower antibacterial activity against anaerobic pathogens can be combined with an anaerobe-killing antimicrobial drug e.g. metronidazole for the management of mixed aerobic/anaerobic infections [2]. This combination is used for the treatment of diarrhea, dysentery and severe perianal Crohn's disease [3].

Patient compliance is critical to the efficacy of therapy regimens as it is the process whereby the patient follows the prescribed regimen as intended by prescriber [4]. The factors that are commonly believed to have an impact on compliance can be categorized into social and psychological domains. These factors include knowledge and understanding, which includes effective communication strategies. Additionally, the quality of the interaction between the patient and the healthcare provider, as well as patient satisfaction, are also influential. Social isolation and social support, including the role of the patient's family, can also affect compliance. Furthermore, health beliefs and attitudes, as well as variables associated with the health belief model, play a significant role. Lastly, factors related to the illness and treatment, such as the complexity and duration of the prescribed regimen, can also influence compliance [5]. The phenomenon of drug interactions holds significant implications for patient adherence or non-adherence to prescribed medication regimens. Enhanced collaboration between general practitioners (GPs) and other healthcare providers is crucial in addressing the challenges associated with polypharmacy. The presence of interactions and unpleasant effects might undermine medication adherence (also known as drug compliance) and self-management, so impeding the attainment of the intended objective. Drug interactions may potentially offer a resolution in situations where the patient's condition has worsened without a definite etiology.Drug interactions play an important role in patient compliance/non-compliance [6].

Several methods for analyzing CIP and MET in their mixture form or alone were discovered in the literature. CIP and MET were determined by spectrophotometric methods [7–20], reversed-phase ion-pair HPLC, TLC-densitometric methods [21], RP-UPLC Technique [22], LC methods [23, 24], UPLC-mass [25], HPLC [26–29] and potentiometric & electrochemical determination [29–32].

No simultaneous techniques were presented for determining the studied drug combination through our introduced methods; advanced absorbance subtraction (AAS), bivariate, spectrum subtraction, and ratio difference methods.

We aimed to create simple, easy, economical, accurate, fast, and uncomplicated techniques for determining the studied drug combinations without additives and/ or excipients interference in the pharmaceutical formulations.

## Experimental

### Apparatus

The Model 6800, a true double beam UV/visible spectrophotometer. Using the Jenway Model 6800 Flight Deck Software.

The measurements were conducted within a 1 cm quartz cell, covering a wavelength range of 200–400 nm, while maintaining the room temperature.

### Materials and reagents

#### Pure standards

Ciprofloxacin and Metronidazole (with purity 99% for both drugs, Batch no.: CPH/16091295 and MT2/3050381 for CIP & MET, respectively) were received as a gift from Aarti Drugs Ltd Co. (Mahendra Industrial Estate, Grand Floor, Road No. 29, Plot No. 109-D, SION (East), MUMBAI-400022. (INDIA).

#### Pharmaceutical formulations

Ciprodiazole® tablets were received from the market (Ciprofloxacin HCl 500 mg and Metronidazole 500 mg) manufactured by MINAPHARM for pharmaceuticals & chemical industries 10th of Ramadan, Egypt.

### Solvents

Distilled water.

### Standard solutions

### Laboratory prepared mixtures

Solutions with varying ratios of CIP and MET were prepared by precisely transferring aliquots from their respective reference solutions into 10 mL volumetric flasks, followed by dilution with distilled water.

### Procedures

#### Construction of calibration curves

For CIP: 1–17 μg/mL working solutions were prepared by the addition of aliquots (0.2, 0.4, 0.5, 0.6, 0.8, 1, 1.2, 1.4, 1.6, 1.8, 2, 2.2, 2.4, 2.6, 2.8, 3, 3.2, 3.4 mL) of CIP working standard solution (50 µg/mL) to 10 mL volumetric flasks series and diluting with distilled water.

For MET: 5–37.5 μg/mL working solutions were prepared by the addition of aliquots (0.2, 0.4, 0.5, 0.75, 1, 1.5, 2, 2.5, 3, 3.5, 4, 4.5, 5, 5.5, 6, 6.5, 7, 7.5 mL) of MET working standard solution (50 µg/mL) to 10 mL volumetric flasks series and diluting with distilled water.

 The absorbance spectra were recorded at ambient temperature in the wavelength range of 200 to 400 nm for all experimental experiments.

#### For advanced absorbance subtraction (AAS) method

This method is done by the subtraction of the amplitude of two equal wavelengths to abolish the effect of one of the drugs; in which one of these wavelengths is an isoabsorptive point from which the total concentration could be calculated.

**Fig. 1:**
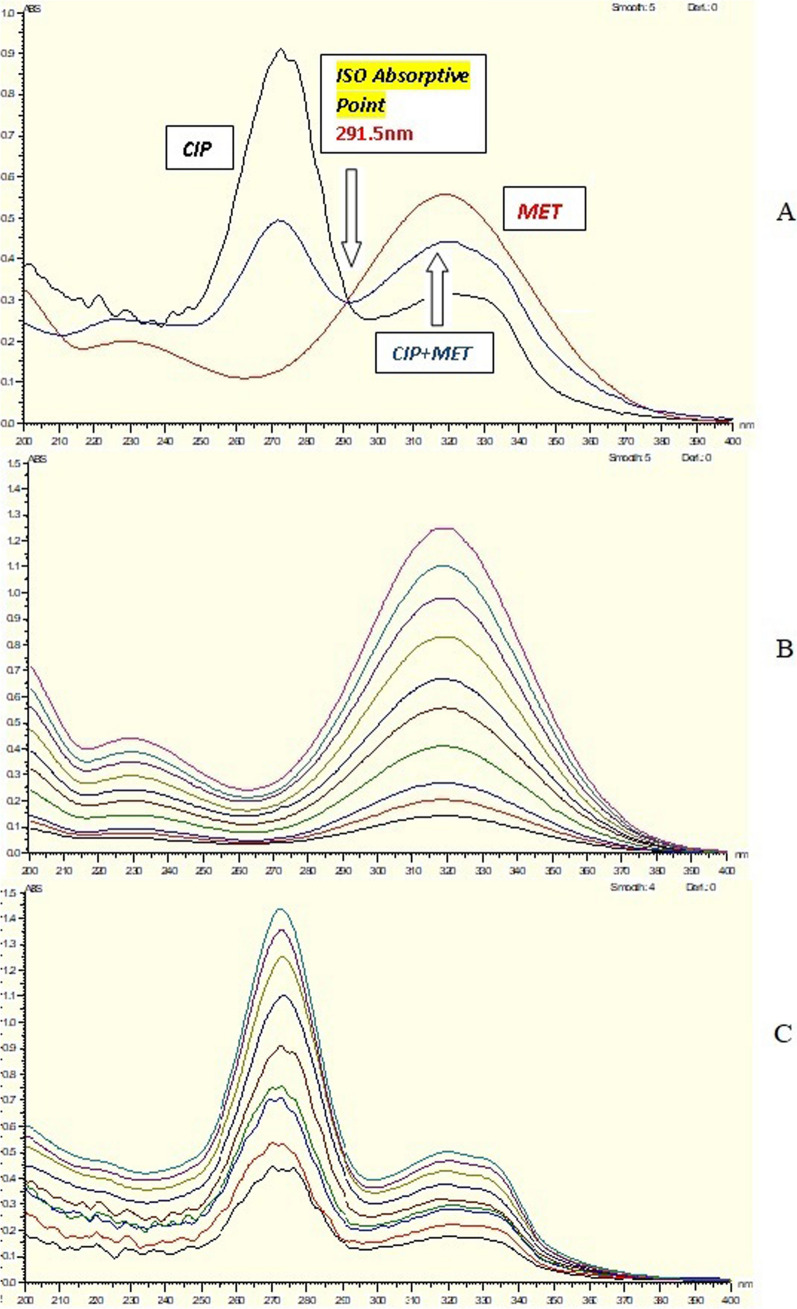
**A** Zero absorption spectrum of 10 µg/mL CIP overlaid with 10 µg/mL MET and a mixture of 5 µg/mL CIP& 5 µg/mL MET revealed that 291.5 nm is an Iso absorptive point and revealed that 291.5 and 250 nm has the same absorbance in CIP and 291.5 nm and 345 nm has the same absorbance in MET, **B** Zero absorption spectra of MET measured at 291.5 nm and 250 nm, and **C** Zero absorption of CIP measured at 291.5 nm and 345 nm

In the presence of CIP, advanced absorbance subtraction (AAS) [33, 34] was employed to calculate MET. The measurement of absorbance was conducted at 291.5 and 250 nm, with 291.5 nm chosen as an isoabsorptive point from which the total concentration could be calculated. Because the wavelengths 291.5 nm and 250 nm have the same absorbance in CIP and the absorbance difference of CIP is zero (Fig. 1A), MET may be calculated using the regression equation (Fig. 1B).

On the other side, CIP was determined in presence of MET. The absorbance was determined at 291.5 and 345 nm, with 291.5 nm chosen as an isoabsorptive point from which the total concentration could be calculated. Because the wavelengths 291.5 nm and 345 nm have the same absorbance in MET and the difference in absorbance of MET is zero (Fig. 1A), CIP may be calculated using the regression equation (Fig. 1C).

### For bivariate method

Two wavelengths were chosen by Kaiser Method for concurrent determination of both drugs by their regression equations.

(A_Ai_ = m_Ai_. C_A_ + e_Ai_) is the formula for the linear calibration regression function used to determine an analyte A at wavelength (i) through spectrophotometry where C is the concentration, m is the slope of the linear regression, and e is the intercept value. We will have two sets of equations if measurements are made for the binary mixture (A and B) at two chosen wavelengths (λ_1_, λ_2_):

$${\text{A}}_{{{\text{AB1}}}} = {\text{m}}_{{{\text{A1}}}} {\text{C}}_{{\text{A}}} + {\text{m}}_{{{\text{B1}}}} {\text{C}}_{{\text{B}}} + {\text{e}}_{{{\text{AB1}}}}$$

$${\text{A}}_{{{\text{AB2}}}} = {\text{m}}_{{{\text{A2}}}} {\text{C}}_{{\text{A}}} + {\text{m}}_{{{\text{B2}}}} {\text{C}}_{{\text{B}}} + {\text{e}}_{{{\text{AB2}}}}$$

As e_AB1_ and e_AB2_ represent the sum of the intercepts at the chosen two wavelengths (e_ABi_ = e_Ai_ + e_Bi_). The C_A_ and C_B_ values could be calculated from the following equations:

$${\text{C}}_{{\text{B}}} = {\text{m}}_{{{\text{A2}}}} \left( {{\text{A}}_{{{\text{AB1}}}} - {\text{ e}}_{{{\text{AB1}}}} } \right) + {\text{m}}_{{{\text{A1}}}} \left( {{\text{e}}_{{{\text{AB2}}}} {-}{\text{A}}_{{{\text{AB2}}}} } \right)/{\text{m}}_{{{\text{AB1}}}} {-}{\text{m}}_{{{\text{A1}}}} {\text{m}}_{{{\text{B2}}}}$$

$${\text{C}}_{{\text{A}}} = {\text{A}}_{{{\text{AB1}}}} {-}{\text{e}}_{{{\text{AB1}}}} {-}{\text{m}}_{{{\text{B1}}}} {\text{C}}_{{\text{B}}} /{\text{m}}_{{{\text{A1}}}}$$

These easy methods could help resolve the binary combination by selecting two wavelengths and using the linear regression parameters to identify each drug under study at the same wavelengths. The Kaiser approach (Table 1) can be used to determine the optimal wavelengths. A series of sensitivity matrices K are computed for every binary mixture and every pair of selected wavelengths:

$${\text{K = }}\left[ {\begin{array}{*{20}c} {{\text{m}}_{{{\text{A1}}}} } & {{\text{m}}_{{{\text{B1}}}} } \\ {{\text{m}}_{{{\text{A2}}}} } & {{\text{m}}_{{{\text{B2}}}} } \\ \end{array} } \right]$$

**Table 1:** Application of the Kaiser method for the selection of wavelength set for the mixture of CIP and MET

λ_2_	λ_1_					
	320 nm	322 nm	324 nm	326 nm	328 nm	330 nm
320 nm	0					
322 nm	11	0				
324 nm	− 24	− 35	0			
326 nm	− 20	− 31	4	0		
328 nm	− 78	− 89	− 53	− 57	0	
330 nm	− 101	− 111	− 76	− 79	− 23	0

**Fig. 2:**
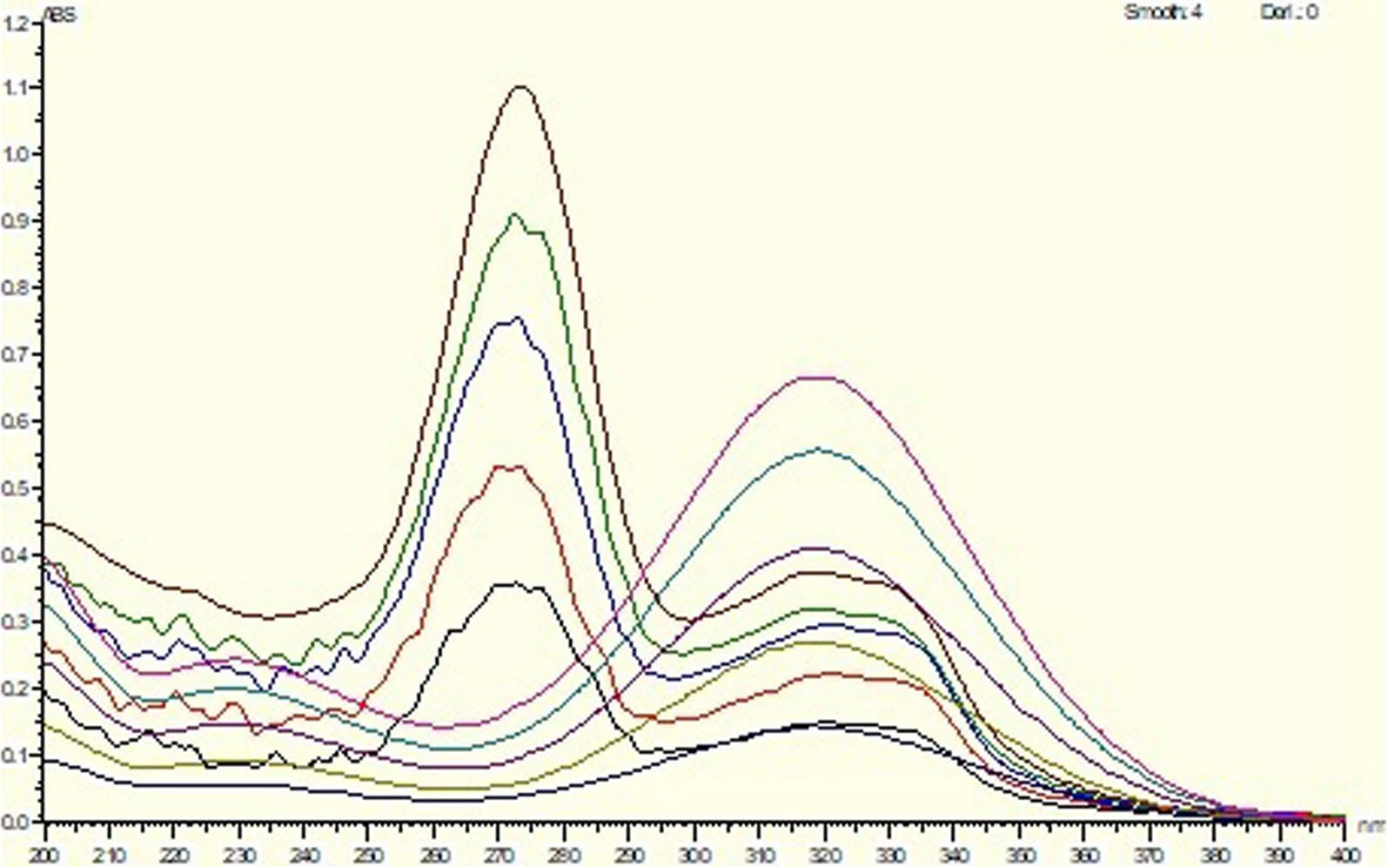
Zero absorption spectra of CIP overlaid with zero absorption spectra of MET

The slopes (sensitivity parameters) of component A are denoted as mA1,2, while the slopes (sensitivity parameters) of component B are denoted as mB1,2. These matrices’ resolution and factors were determined. The set of wavelengths that has the highest absolute matrix determinant was chosen [35]. The bivariate technique was employed to determine CIP and MET in the presence of each other at the same wavelengths (322 and 330 nm) (Fig. 2).

### For ratio difference method

This method is done by subtraction of the resultant amplitudes from the division of the spectra of the drug by a divisor from the second drug to abolish its effect in results.

**Fig. 3:**
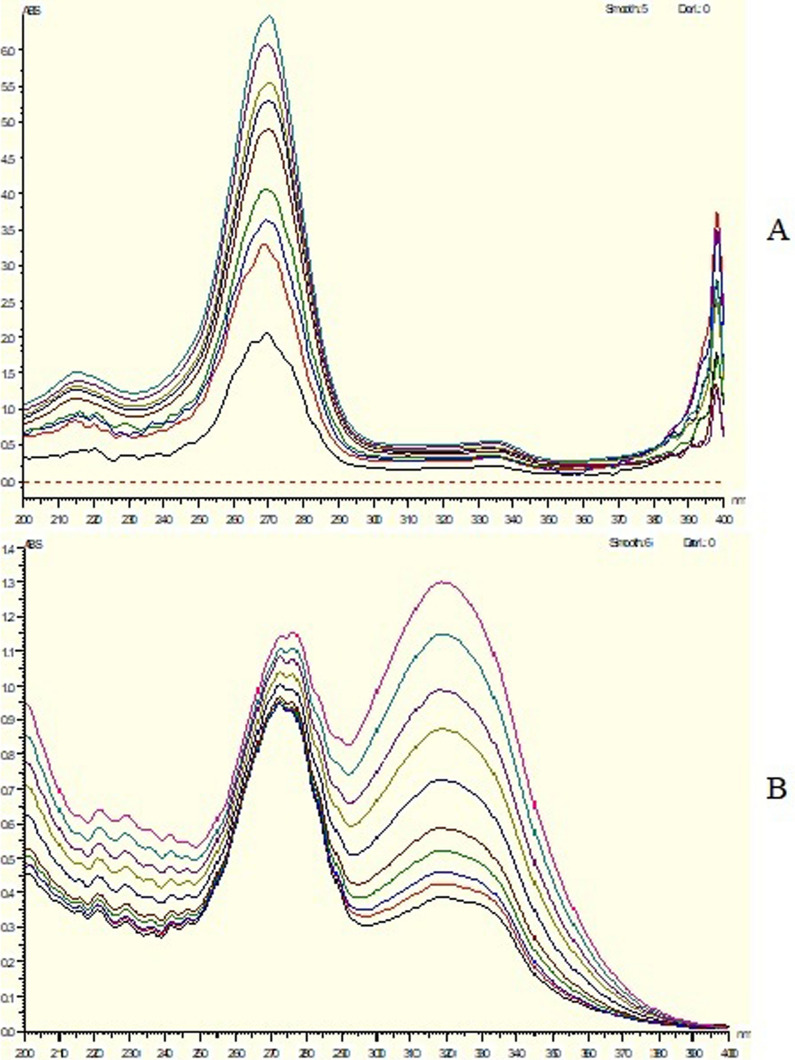
Ratio spectra of **(A)** CIP using 17.5µg/mL MET as a divisor, **(B)** MET using 10µg/mL CIP as a divisor

CIP and MET were calculated by utilizing ratio difference approach [36, 37]. CIP and MET: The CIP zero absorption spectra were first captured and preserved, then divided by 17.5 μg/mL MET spectrum (divisor) in which is the best one from several trials. At 260 and 270 nm (the best wavelengths from trials), the resultant spectra's amplitudes were measured. The difference was calculated and utilized to generate the calibration curve (Fig. 3A). The MET zero absorption spectra, on the other hand, were recorded and saved before being split by the spectrum of 10 μg/mL CIP (divisor). The generated spectra's amplitudes were measured at 320 and 360 nm. The difference between them was calculated and utilized to create the calibration curve (Fig. 3B), after which the regression equation calculations were performed.

### For spectrum subtraction method

The zero spectra of the desired drug is obtained by subtracting the spectra of one drug from the spectra of the mixture.

Spectrum Subtraction method [38, 39] was used in CIP determination in presence of MET. Absorbance was calculated at wavelength 271.5 nm (Fig. 1C) which is the maximum wavelength of CIP after subtraction of MET spectra from laboratory mixture of two drugs. On the other hand, MET was determined in presence of CIP Absorbance was calculated at 321 nm (Fig. 1B) which is the maximum wavelength of MET after subtraction of CIP spectra from laboratory mixture of two drugs.

### Analysis of laboratory prepared mixtures

After generating different ratios of laboratory-prepared mixtures, the spectra of these mixtures were evaluated and processed using the suggested methodologies.

### Application to pharmaceutical formulation

Following the weighting and crushing of 10 Ciprodiazole® tablets, a quantity equal to each tablet (500 mg CIP and 500 mg MET) was diluted with distilled water in a 50 mL volumetric flask as follows:

Before filtering, 30 mL of pure distilled water were added, sonicated, and diluted to the proper concentration. Second, 1 mL of the dilution was placed in a 100 mL volumetric flask to achieve a concentration of 100 μg/mL CIP and 100 μg/mL MET. Third, any additional dilutions were performed in 10 mL volumetric flasks and handled in accordance with the proposed methods.

### Comparative study

Comparative study between the 4 proposed methods (Spectrum Subtraction, Advanced absorbance Subtraction, Bivariate and Ratio Difference) in terms of sensitivity, specificity, and ease of manipulation is performed and introduced in Table 5.

## Results and discussion

### Spectrum subtraction

At 271.5 nm, where CIP can be estimated in the presence of MET, absorbance was measured. With a correlation coefficient of 0.9991, the calibration curves showed established linear correlations between concentrations and the spectrum subtraction in the range of 2–17 μg/mL for CIP. The method's accuracy revealed that approved values fell within 98.06% ± 0.02. Additionally, the method specificity revealed that values 100.07% ± 1.59 were accepted. The results are demonstrated in Table 2.

**Table 2:** Assay parameters and validation results obtained by applying the spectrum subtraction, advanced absorbance subtraction, bivariate and ratio difference spectrophotometric methods

Method parameters	Spectrum Subtraction spectrophotometric method		Advanced absorbance Subtraction spectrophotometric method		Bivariate spectrophotometric method				Ratio Difference spectrophotometric method	
	Determination of CIP in presence of MET	Determination of MET in presence of CIP	Determination of CIP in presence of MET	Determination of MET in presence of CIP	Determination of CIP in presence of MET		Determination of MET in presence of CIP		Determination of CIP in presence of MET	Determination of MET in presence of CIP
Wavelength (nm)	271.5	321	291.5–345	251–316	322	330	322	330	260–270	360–320
Linearity range (µg/mL) (n = 3)	2–17	5–37.5	3–16	1–37.5	3–19	3–19	2–35	2–35	1–16	1–37.5
Intercept	0.0162	− 0.0454	− 0.0710	− 0.0216	0.0290	0.0334	− 0.0228	− 0.0124	− 0.0013	0.0645
Slope	0.0895	0.0580	0.0237	0.0437	0.0289	0.0267	0.0570	0.0499	0.1276	0.1232
Correlation coefficient (r)	0.9991	0.9994	0.9996	0.9994	0.9994	0.9993	0.9992	0.9994	0.999	0.999
Accuracy (Mean ± SD)	98.06 ± 0.02	100.08 ± 0.02	101.27 ± 1.10	100.28 ± 0.83	99.77 ± 0.74	99.63 ± 0.91	99.50 ± 0.97	99.13 ± 0.33	100.75 ± 0.62	98.93 ± 0.31
Precision (± %RSD)										
Repeatability	101.04 ± 1.5	100.42 ± 1.21	101.47 ± 1.13	99.77 ± 1.32	101.10 ± 1.28	99.61 ± 0.92	100.61 ± 0.75	100.59 ± 0.65	100.39 ± 0.47	99.34 ± 0.35
Intermediate precision	98.44 ± 1.46	101.22 ± 1.45	101.79 ± 1.32	100.09 ± 1.09	101.02 ± 0.87	99.73 ± 1.04	101.16 ± 0.69	101.10 ± 0.65	101.04 ± 0.81	99.56 ± 0.38
Specificity (Mean ± SD)	100.07 ± 1.59	99.16 ± 1.54	99.34 ± 1.52	99.46 ± 1.22	99.97 ± 1.47		100.02 ± 1.45		100.10 ± 1.48	99.79 ± 0.95

In contrast, the absorbance was determined at 321 nm, where MET can be calculated when CIP is present. As the correlation coefficient is equal to 0.9994, the calibration curves exhibited that there are linear connections between concentrations and the dual wavelength in the range of 5–37.5 μg/mL for MET. The method's accuracy revealed that approved values fell within the range of 100.08% ± 0.02. Additionally, the technique specificity revealed that approved values fell between 99.16% ± 1.54. The results are demonstrated in Table 2. Spectrum subtraction is very easy and simple as spectrum subtraction doesn't require any additional processing because it relies on zero absorption spectra. It just requires a few steps to obtain the necessary drug's zero order spectra, but noise interference prevents it from achieving the desired drug concentration by subtraction.

### Advanced absorbance subtraction (AAS) method

Advanced absorbance subtraction (AAS) absorbance measurements were made at 291.5 and 345 nm for CIP and 291.5 and 250 nm for MET, with 291.5 nm chosen as the iso-absorptive point (Total Conc.) at which CIP and MET could both be assessed in the existence of the other. CIP concentrations had a correlation value of 0.9996 and were linear in the 3–16 μg/mL range. The technique displayed acceptable specificity values of 99.34% ± 1.52 and acceptable accuracy values of 101.27% ± 1.10 (Table 2). On the other hand, MET concentrations had a correlation value of 0.9994 and were linear in the range of 1–37.5 g/mL. The technique displayed acceptable specificity values of 99.46% ± 1.22 and acceptable accuracy values of 100.28% ± 0.83. (Table 2). Advantages and restrictions of advanced absorbance subtraction (AAS) method are the similar as the previous method.

### Bivariate method

The absorbance values were determined at 322 and 330 nm, as CIP and MET may both be evaluated using the same wavelengths. The calibration curves demonstrated acceptable linear correlations between concentrations and the bivariate in the 3–19 μg/mL range for CIP and 2–35 μg/mL range for MET, with correlation coefficients of 0.9994 and 0.9993 for CIP and 0.9992 & 0.9994 for MET. The method's accuracy showed approved values of 99.77% ± 0.74 & 99.63 ± 0.91 for CIP and 99.5% ± 0.97 & 99.13 ± 0.33 for MET. The method specificity revealed accepted values with 99.97% ± 1.47 for CIP and 100.02% ± 1.45 for MET. The results are documented in Table 2. Bivariate, as the techniques discussed previously, is very basic, accurate, and simple.

The only limitation of this strategy is that it requires applying the Kaiser method before selecting the two ideal wavelengths.

### Ratio difference method

Using 17.5 μg/mL MET as a divisor, absorbance readings at 260 and 270 nm were used to determine CIP. On the other hand, absorbance at 320 and 360 nm was measured to estimate MET using 10 μg/mL CIP as a divisor. The calibration curves showed established linear relationships between concentrations and the ratio difference in the range of 1–16 μg/mL for CIP and 1–37.5 μg/mL for MET, with correlation coefficients of 0.999 for both. Acceptable values of 100.75 ± 0.62 for CIP and 98.93 ± 0.31 for MET were found by the method accuracy. Additionally, approved values of 100.10% ± 1.84 for CIP and 99.79% ± 0.095 for MET were revealed by the method specificity. Table 2 illustrates the results. The ratio difference strategy is a simple and precise technique, as was previously indicated because the method does not rely on any special software. The drawback of this method is that it necessitates splitting the target compound's spectrum by a particular divisor of the other drug and conducting numerous tests to determine which divisor works best.

### Method validation

According to ICH standards, each technique was validated [40]. The calibration curves' linear regression data revealed solid linear relationship (Table 2).

As demonstrated in Table 2, the accuracy was determined by examining one concentration 3 times, which yielded satisfactory findings.

By testing the laboratory-prepared MET & CIP mixtures within the linearity range, the specificity of the techniques was determined, and positive findings were achieved (Table 2).

The study of 3 different medication concentrations 3 times on the same day and 3 consecutive days allowed for the calculation of the intra- and inter-day precisions (Table 2).

### Analysis of laboratory prepared mixtures

To calculate CIP and MET in varied concentrations within the linearity range in Laboratory prepared mixtures, the suggested procedures were successfully applied and positive findings were achieved revealing no interference of each drug in presence of the other when applying the proposed methods (Table 3).

**Table 3:** Analysis of the Laboratory Mixture of Ciprofloxacin and Metronidazole by applying the bivariate, ratio difference, advanced absorption subtraction and spectrum subtraction spectrophotometric methods

	Bivariate method				Ratio difference method				Advanced absorption subtraction				Spectrum subtraction			
	CIP		MET		CIP		MET		CIP		MET		CIP		MET	
	WL (322–330)	Recovery%	WL (322–330)	Recovery%	WL (270–260)	Recovery%	WL (320–360)	Recovery%	WL (291.5–345)	Recovery%	WL (316–251)	Recovery%	WL (271.5)	Recovery%	WL (321)	Recovery%
	Conc	Rec. %	Conc	Rec. %	Conc	Rec. %	Conc	Rec. %	Conc	Rec. %	Conc	Rec. %	Conc	Rec. %	Conc	Rec. %
	5	98.72	5	101.32	7	99.01	1	100.23	10	101.87	5	101.48	2.5	98.74	2.5	101.67
	7	101.85	7	99.61	10	98.94	2	99.62	11	99.43	8	99.64	5	101.31	5	99.13
	7.5	99.32	7.5	99.56	13	101.44	3	99.98	12	98.31	9	98.84	7.5	101.69	7.5	99.73
	11	98.72	11	98.03	14	98.33	4	100.84	12.5	98.02	12.5	98.93	10	98.17	10	99.15
	12	101.22	12	101.56	15	101.38	5	98.29	13	98.08	13	98.41	15	101.50	15	97.11
					16	101.48			15	99.76	15	99.48	17	99.03	20	98.16
Mean		99.97		100.02		100.10		99.79		99.34		99.46		100.07		99.16
SD		1.47		1.45		1.48		0.95		1.52		1.22		1.59		1.54

### Application to pharmaceutical formulation

To determine CIP & MET in pharmaceutical formulations (Ciprodiazole® tablets), the suggested procedures were successfully applied. The outcomes were respectable and sufficiently in line with the amounts indicated on the labels. There was no excipient interference, according to the results of the standard addition procedure (Table 4).

**Table 4:** Analysis of the pharmaceutical preparation (Ciprodiazole® tablets) by applying the bivariate, ratio difference, advanced absorption subtraction and spectrum subtraction spectrophotometric methods

	Bivariate method								Ratio difference method							
	CIP				MET				CIP				MET			
			Recovery%				Recovery%				Recovery%				Recovery%	
	Tablet	Standard added (µg/mL)	Tablet	Added	Tablet taken (µg/mL)	Standard added (µg/mL)	Tablet	Added	Tablet taken (µg/mL)	Standard added (µg/mL)	Tablet	Added	Tablet taken (µg/mL)	Standard added (µg/mL)	Tablet	Added
	5	1	98.72	100.70	5	1	101.32	100.71	5	1	98.19	98.78	5	1	100.67	102.06
		6	98.55	98.06		6	101.38	98.39		6	98.51	101.25		6	101.13	99.74
		10	100.66	99.16		10	100.16	98.26		10	100.86	99.02		10	101.60	100.83
Mean			99.31	99.31			100.96	99.12			99.18	99.68			101.13	100.87
SD			1.17	1.32			0.69	1.38			1.46	1.36			0.47	1.16

	Advanced absorption subtraction								Spectrum subtraction							
	CIP				MET				CIP				MET			
			Recovery%				Recovery%				Recovery%				Recovery%	
	Tablet taken (µg/mL)	Standard added (µg/mL)	Tablet	Added	Tablet taken (µg/mL)	Standard added (µg/mL)	Tablet	Added	Tablet taken (µg/mL)	Standard added (µg/mL)	Tablet	Added	Tablet taken (µg/mL)	Standard added (µg/mL)	Tablet	Added
	5	1	100.22	101.41	5	1	99.21	100.35	5	1	101.31	100.76	5	1	99.13	99.51
		6	99.96	100.3		6	98.26	98.78		6	99.08	98.69		6	98.09	100.26
		10	100.55	102.48		10	101.11	100.95		10	99.52	101.52		10	99.82	99.5
Mean			100.25	101.41			99.53	100.03			99.97	100.32			99.01	99.76
SD			0.3	1.09			1.45	1.12			1.18	1.46			0.87	0.44

### Comparative study

Comparative study between the 4 proposed methods (Spectrum Subtraction, Advanced absorbance Subtraction, Bivariate and Ratio Difference) in terms of sensitivity, specificity, and ease of manipulation is performed; in which ratio difference is the most sensitive, bivariate is the most specific and advanced absorbance subtraction is the easiest method and all of the comparison is introduced in Table 5.

**Table 5:** Comparative study between bivariate, ratio difference, advanced absorption subtraction and spectrum subtraction spectrophotometric methods in terms of sensitivity, specificity, and ease of manipulation

Method parameters	Spectrum subtraction spectrophotometric method	Advanced absorbance subtraction spectrophotometric method	Bivariate spectrophotometric method	Ratio difference spectrophotometric method
Sensitivity	The least sensitive	Sensitive	Sensitive	The most sensitive
Specificity	Specific	The least specific	The most specific	More specific than spectrum subtraction
Ease of Manipulation	Easy method	The easiest method	Easy method	The most difficult

### Statistical analysis

The software PASW statistics 18® was used to do a one-way ANOVA statistical comparison of the suggested strategies. The computed F values were found to be lower than the expected values, indicating that there is no significant difference between the stated methodology and the recommended one [7] (Table 6).

**Table 6:** Statistical comparison of the results obtained by the proposed methods using One-way ANOVA

Tablets	Drugs		Sum of squares	df	Mean square	F	Sig
Ciprodiazole® tablets	CIP	Between groups	3.442	4	0.861	0.629	0.653
		Within groups	13.688	10	1.369		
		Total	17.130	14			
	MET	Between groups	6.846	4	1.711	2.695	0.093
		Within groups	6.350	10	0.635		
		Total	13.196	14			

## Conclusion

Ciprofloxacin and Metronidazole in their combination medicinal dose forms were determined using bivariate, advanced absorbance subtraction (AAS), ratio difference and spectrum subtraction methods. All of the suggested procedures can be successfully applied for routine analysis with the aid of low-tech equipment or technology since they are simple, straightforward, accurate, and sensitive. By comparing of the prior ways, it was shown that ratio difference only needs additional processing while spectrum subtraction, advanced absorbance subtraction (AAS), and bivariate methods do not. Comparative study between the 4 proposed methods is performed in which ratio difference is the most sensitive, bivariate is the most specific and advanced absorbance subtraction is the easiest method. The proposed methods are more accurate, specific and sensitive than previously spectrophotometric methods. The proposed methodologies and the reported method did not significantly differ, according to statistical analysis.

### Supplementary Information

**Additional file 1: Fig. S1.** Chemical structures of Ciprofloxacin (CIP) & Metronidazole (MET).

## Data Availability

The authors confirm that the data supporting the findings of this study are available within the article [and/or] its Additional file.
