# Supplementary material for: Different methods for resolving overlapping UV spectra of combination medicinal dose forms of ciprofloxacin and metronidazole
Source: BMC Chem. 2023 Oct 10;17(1):137. doi: 10.1186/s13065-023-01007-z (PMC10566196; doi:10.1186/s13065-023-01007-z)
Supplement: Supplementary file 1 — Additional file 1: Fig. S1. Chemical structures of Ciprofloxacin (CIP) & Metronidazole (MET). [file 13065_2023_1007_MOESM1_ESM.docx]

Fig. S1: Chemical structures of Ciprofloxacin (CIP) & Metronidazole (MET).
